# Supplementary material for: Report of Adverse Effects Following Population-Wide COVID-19 Vaccination: A Comparative Study between Six Different Vaccines in Baja-California, Mexico
Source: Vaccines (Basel). 2022 Jul 27;10(8):1196. doi: 10.3390/vaccines10081196 (PMC9414877; doi:10.3390/vaccines10081196)
Supplement: Supplementary file 1 [file vaccines-10-01196-s001.zip › vaccines-1788510-supplementary.pdf]

Vaccine platform	Vaccine manufacturer	Clinical Trials data			Baja-California data		
		Administered doses	Reported AE (n)	AE per 100,000 doses	Administered doses	Reported AE (n)	AE per 100,000 doses
mRNA	Pfizer-BioNTech	21,621 [38]	5770	26,687.01	1,282,792	1,014	79.05
Viral vector	CanSinoBIO	18,363 [36]	442	2,407.01	959,990	81	84.38
	Johnson & Johnson	3,356 [35]	440	13.11	1,256,494	825	65.66
	AstraZeneca	21,587 [37]	8771	40,630	608,547	214	35.71
	Serum Institute of India	1200 [31]	602	50,166.66	23,810	113	474.59
Inactivated virus	Sinovac	5,350,038 [34]	2,584	48.29	248,761	38	15.28
	Total	*5,416,165*	*18,609*	*343.58*	*3,516,394*	*2,285*	*64.98*

Table S1. Adverse events rates per 100,000 doses reported in Clinical Trials for SARS-CoV-2 vaccines compared with those reported in the Epidemiological Surveillance System of Baja California, Mexico

Vaccine platform	Vaccine manufacturer	Population-based studies data			Baja-California data		
		Administered doses	Reported AE (n)	AE per 100,000 doses	Administered doses	Reported AE (n)	AE per 100,000 doses
mRNA	Pfizer-BioNTech	27,000,000 [43]	171,913	636.71	1,282,792	1,014	79.05
Viral vector	CanSinoBIO	575,152 [44]	216	37.55	95,9990	81	84.38
	Johnson & Johnson	7,980,000 [19]	13,725	171.99	1,256,494	825	65.66
	AstraZeneca	24,900,000 [43]	245,771	987.03	608,547	214	35.71
	Serum Institute of India	21,115 [45]	201	951.92	23,810	113	474.59
Inactivated virus	Sinovac	20,839,269 [44]	6,440	30.9	248,761	38	15.28
	Total	*81,315,536*	*438,266*	*538.96*	*3,516,394*	*2,285*	*64.98*

Table S2. Adverse events rates per 100,000 doses reported in population-based reports and studies reporting adverse events following vaccination against SARS-CoV-2 compared with those reported in the Epidemiological Surveillance System of Baja California, Mexico

Baseline characteristics	AZ SII n=113	Other vaccines n=2172	p-value
Age - mean ± SD	50.06 ± 11.94	36.84 ± 12.84	<0.0001
Women - n (%)	76 (67.25)	1456 (67.03)	0.99
Pregnant - n(%)	4 (5.26)	25 (1.71)	0.06
History of allergic reaction - n(%)	13 (11.5)	313 (14.41)	0.46
Food	0 (0)	45 (2.07)	0.23
Drugs	9 (7.96)	172 (7.9)	0.99
Polen	0 (0)	26 (1.19)	0.47
Ignores	0 (0)	8 (0.36)	0.99
Other	4 (3.53)	24 (1.1)	0.06

Table S3. Post-hoc analysis comparing demographic characteristics and history of allergies between individuals who developed AE in the AZ SII group vs all other vaccines. These analyses are explorative and were conducted after the observed differences between the rates of AE in the SII group compared to the other manufacturers. The present study may not be equipped to make more conclusions about these findings in terms of having enough statistical power to assess these post-hoc analyses.
